# The value of 3D pseudo-continuousarterial spin labeling perfusion imaging in moyamoya disease—Comparison with dynamic susceptibility contrast perfusion imaging

**DOI:** 10.3389/fnins.2022.944246

**Published:** 2022-08-05

**Authors:** Hongtao Zhang, Mingming Lu, Shitong Liu, Dongqing Liu, Xuxuan Shen, Fugeng Sheng, Cong Han, Jianming Cai

**Affiliations:** ^1^Department of Radiology, The Fifth Medical Center of Chinese People’s Liberation Army (PLA) General Hospital, Beijing, China; ^2^Department of Radiology, Pingjin Hospital, Characteristic Medical Center of Chinese People’s Armed Police Force, Tianjin, China; ^3^Department of Neurosurgery, The First Medical Center of Chinese People’s Liberation Army (PLA) General Hospital, Beijing, China

**Keywords:** magnetic resonance imaging, arterial spin labeling, dynamic susceptibility contrast enhanced perfusion, moyamoya disease, cerebral blood flow

## Abstract

**Background and purpose:**

3D pseudo-continuous arterial spin labeling (3D pCASL) is commonly used to measure arterial cerebral blood flow (CBF). The aim of this study was to assess the clinical feasibility and accuracy of 3D pCASL in comparison with dynamic susceptibility contrast (DSC) perfusion imaging in moyamoya disease (MMD).

**Materials and methods:**

A total of 174 MMD patients underwent 3D pCASL and DSC-MRI for evaluating cerebral blood perfusion. 3D-pCASL with two single post-labeling delay (PLD) times (1,500 and 2,500 ms) was used to measure CBF. The values of DSC-CBF and ASL-CBF were calculated for major arterial territories including the anterior, middle, and posterior cerebral arteries as well as the areas based on the Alberta Stroke Program Early CT Score (ASPECTS) template. The correlation between DSC-CBF and ASL-CBF was analyzed. The consistency and accuracy between the two methods in assessing the cerebral ischemic state before and after surgery were analyzed.

**Results:**

The correlation between ASL (2,500 ms) and DSC-MRI was slightly better than the correlation between ASL (1,500 ms) and DSC-MRI in major vascular territories before revascularization. Significant correlations were observed between ASL (2,500 ms) and DSC-MRI and between ASL (1,500 ms) and DSC-MRI in major vascular territories after revascularization. For 44 surgically treated patients, the scores of ASPECTS for CBF on the operated side were significantly different before and after revascularization (*p* < 0.05) and showed good consistency on all the examination methods. A comparison of the scores of ASPECTS of the three parameters before and after revascularization showed that there was no statistical difference between them (*p* > 0.05).

**Conclusion:**

Compared to DSC-MRI, 3D pCASL can assess the cerebral blood perfusion in MMD before and after revascularization effectively. 3D pCASL showed the feasibility and clinical utility value in patients with MMD.

## Introduction

Moyamoya disease (MMD) is a chronic cerebrovascular disease characterized by gradual intimal thickening and progressive narrowing or occlusion at the junction of the bilateral internal carotid artery and the common origin of the middle cerebral artery and the anterior cerebral artery and is associated with compensatory expansion and stenosis of perforating arteries in the Circle of Willis (Qiao et al., 2017; Bang et al., 2020). The cerebral hemodynamics of patients with MMD is very complex. Therefore, for patients with MMD, an accurate assessment of cerebral hemodynamics is very important for the selection of treatment and evaluation of efficacy (Ishii et al., 2014, 2017).

Dynamic susceptibility contrast (DSC) perfusion imaging is an magnetic resonance imaging (MRI) technique that allows multiparameter assessment of cerebral hemodynamics. Compared with PET, the standard examination method of cerebral perfusion, MRI has no radioactivity and has better clinical availability, so DSC is widely used in the evaluation of cerebrovascular diseases (Wintermark et al., 2005; Zhang et al., 2018). The main disadvantages of DSC-MRI are the need for injection of contrast and semi-quantitative assessment of the parameters.

In the last decade, the use of arterial spin labeling (ASL) to quantify cerebral perfusion (in units of ml/100 g/min) has become increasingly popular (Haller et al., 2016; Kohno et al., 2016; Havsteen et al., 2018; Shao et al., 2018; Hu et al., 2019), as ASL is a non-invasive and reproducible examination that does not apply to contrast agents. In the classic application of 3D pseudo-continuous arterial spin labeling (3D pCASL), a single PLD is used. The PLD represents the time between the blood labeling by radiofrequency pulses, usually at the carotid artery level, and the time when 3D data acquisition begins with the imaging volume. In the study by Alsop et al. (2015) and Nery et al. (2018), a PLD of 2,000 ms was recommended for neonates, 1,500 ms for children, and 1,800 ms for adults. However, there is no clear recommendation for PLD in MMD patients. The purpose of our study was to observe the collateral circulation in the brain of patients with MMD, so two delay times were adopted, which were 1,500 and 2,500 ms.

The aim of this research was to study the correlation between dynamic susceptibility contrast-cerebral blood flow (DSC-CBF) and ASL-CBF using two single PLDs of 1,500 and 2,500 ms in patients with MMD. The results of the scores of ASPECTS of DSC-CBF and ASL-CBF were compared before and after revascularization to assess the value of changes in cerebral blood flow (CBF) in MMD for ASL.

## Materials and methods

### Patients

This was a prospective study and this study was approved by our Institutional Review Board. Informed consent was obtained from all patients. Between October 2020 and September 2021, 174 patients with angiographically confirmed MMD were included in the study. All patients underwent DSC and ASL examination before surgery. A total of 44 of these patients underwent encephaloduroarteriosynangiosis (EDAS) and DSC and ASL examinations were performed 3–4 months after surgery. The inclusion criteria were as follows: (1) patients diagnosed with MMD by digital subtraction angiography (DSA) according to the current diagnostic criteria and who underwent revascularization surgery for evaluation; (2) patients with preoperative and postoperative MRI data (patients who underwent DSC-MRI, 3D pCASL, and T1WI 3D-MPRAGE before and after surgery). The exclusion criteria were as follows: (1) secondary moyamoya phenomenon caused by other well-diagnosed diseases; (2) brain tumors; (3) intracranial aneurysms, and; (4) contradiction to MR examination.

### Magnetic resonance imaging protocols

MR imaging examinations were performed with a 3.0-T system (MAGNETOM SKYRA, Siemens Healthcare) with a 20-channel head coil. The scan sequences included DSC-MRI, ASL-MRI, and T1WI 3D MPRAGE. T1WI 3D-MPRAGE and 3D pCASL were scanned together and DSC-MRI was done the next day.

DSC-MRI, the axial brain MR imaging parameters were as follows: repetition time (TR)/echo time (TE) = 1,360/30 ms, slice thickness = 5 mm, intersection gap = 1.5 mm, field of view (FOV) = 230 × 230 mm^2^, matrix size = 144 × 144, number of excitation (NEX) = 1. A gadolinium contrast agent of gadolinium-diethylenetriaminepentaacetic acid (Gd-DTPA) was injected intravenously at a rate of 4∼4.5 ml/s (20 ml) using a power injector at the sixth acquisition, followed by a 30-ml saline flush. The DSC-MRI original images obtained were processed by a post-processing workstation (Syngo *Via* 20, Simens) and analyzed using brain MRI perfusion software. Four parameters [relative cerebral blood volume (CBV), relative CBF, relative mean transit time (MTT), and time to peak (TTP)] were automatically obtained.

The ASL scan used a pCASL pulse sequence with background suppressed 3D Gradient and Spin Echo (GRASE) readout labeling pulse duration = 1,500 ms, PLD = 1,500 and 2,500 ms, no flow crushing gradient, FOV = 224 × 224 mm^2^, matrix size = 64 × 64, TE/TR = 36.76/4,000 ms, thirty-two 4-mm slices acquired for covering the whole brain.

For structural imaging, T1-weighted sagittal images were acquired with a 3D MPRAGE sequence. The parameters were as follows: 192 slices at 1 mm slice thickness, voxel size = 1 × 1 × 1 mm^3^, TR/TE = 2,000/2.26 ms, and the scan time of 4 min and 40 s.

### Image analysis

The CBF value was automatically measured by software as follows: (1) ASL image data were processed using CereFlow software and were used to automatically calculate the value of CBF. The DSC-MRI images acquired were analyzed using the MR Neuro-Perfusion software (Syngo *Via* 20, Simens), automatically generating relative CBV, relative CBF, relative MTT, and TTP, with (2) co-registration with the high-resolution sagittal anatomical T1WI image and (3) standardization of T1WI anatomical images to the Montreal Neurological Institute (MNI) template. (4) The CBF image warped into MNI space using the forward transformation matrix derived from T1WI and regional CBF was extracted by applying the ASPECTS and vascular territory atlases, as was applied in the previous study by Wang and his colleagues (Tatu et al., 1998; Wang et al., 2013).

The quantitative parameters of DSC and ASL for major arterial territories of anterior cerebral artery (ACA), middle cerebral artery (MCA), posterior cerebral artery (PCA), and cerebellar hemispheres were measured in the bilateral cerebral hemispheres in all 174 patients with MMD. CBF was measured in the region of interests (ROIs) of DSC and ASL parameter maps. The values of the ratio of CBF of the supply territories of ACA, MCA, and PCA to ipsilateral cerebellar hemispheres were calculated (CBF_relative_ = CBF_vascular territories_/CBF_cerebellar hemispheres_). For 44 surgically treated patients, CBF values of the supply territories of ACA, MCA, PCA, and cerebellar hemispheres before and after revascularization were measured on the surgical site. The values of the ratio of CBF of the supply territories of ACA, MCA, and PCA to ipsilateral cerebellar hemispheres were calculated (CBF_relative_ = CBF_vascular territories_/CBF_cerebellar hemispheres_). The values of the difference between the postoperative ratios of CBF and preoperative ratios of CBF were calculated (CBF_minus_ = postoperative CBF_relative_-preoperative CBF_relative_). Finally, the correlations between DSC-CBF_relative_ and ASL-CBF_relative_ and between DSC-CBF_minus_ and ASL-CBF_minus_ were analyzed.

Evaluation of cerebral ischemic state: The assessment of the ischemic status of ASPECTS areas involved a quantitative assessment of ASL-CBF. The CBF values of ASPECTS areas less than 30 ml/100 g/min were defined as ischemic states. The assessment of the ischemic status of ASPECTS areas involved a qualitative assessment of DSC-CBF. The diagnostic criteria for ischemia were the presence of a decreased signal on the DSC-CBF images and the increased signal on the TTP images compared to the normal cerebral vascular area. DSC-CBF was independently evaluated by two experienced neuroradiologists and used to assess the presence of ischemia on ASPECTS areas. The final decisions were reached by consensus. The areas of ASPECTS standards assessment include 10 areas of MCA-related blood supply area. The normal ASPECTS area (without ischemia) scored 1 point, and the ischemic area scored 0 points. All ASPECTS areas without ischemic change scored 10 points, and all ASPECTS areas with ischemic change scored 0 points. The consistency and change between DSC and ASL in assessing preoperative and postoperative scores of ASPECTS were also analyzed.

### Statistical analysis

The statistical analysis was performed using SPSS (version 22.0, IBM). All reported *p-*values were two-sided and were considered statistically significant at values of less than 0.05. According to the results of the normality test, the correlations between DSC-CBF and ASL-CBF were counted using Spearman’s correlation analysis. The consistency between ASL-CBF (PLD = 1,500 and 2,500 ms) and DSC-CBF in assessing preoperative and postoperative ASPECTS scores was examined by calculating the intra-class correlation coefficient (ICC). A one-way ANOVA was used for assessing preoperative and postoperative ASPECTS scores of ASL-CBF (PLD = 1,500 and 2,500 ms) and DSC-CBF. Improvement of ASPECTS scores of postoperative and preoperative ischemic state was examined by *t*-test.

## Results

### Demographic and clinical information

A total of 174 patients with MMD underwent simultaneous preoperative DSC and ASL (PLD = 1,500 and 2,500 ms). The mean age of patients was 41.1 years (range: 14–66 years, 89 men). Forty-four of these patients underwent EDAS on one side. The mean age of surgical patients was 39.5 years (range: 15–60 years, 22 men) (see Table 1). All postoperative patients also underwent simultaneous preoperative DSC and ASL and followed by MRI examination 3–4 months after revascularization. CBF was measured in major arterial territories of ACA, MCA, and PCA in the bilateral cerebral hemispheres in all 174 patients, as well as in the corresponding cerebellar hemispheres. CBF values of all ASPECTS areas in the cerebral hemispheres before and after revascularization were measured.

**TABLE 1:** The clinical and imaging characteristics of the patients with moyamoya disease (MMD).

Characteristics		All 174 MMD patients	44 Surgical patients
Age (years)		41.1 ± 11.1	39.5 ± 10.6
Male/Female (*n*)		89/85	22/22
Hemorrhagic stroke (*n*)		16 (9.2%)	3 (6.8%)
	Frontal lobe	121 (69.5%)	31 (70.5%)
	Parietal lobe	58 (33.3%)	16 (36.4%)
Ischemic stroke (*n*)	Occipital lobe	25 (14.4%)	6 (13.6%)
	Temporal lobe	23 (13.2%)	5 (11.4%)
	Basal ganglia	42 (24.1%)	4 (9.1%)

(n) is the number of patients. Other data are mean values ± standard deviations.

### Quantitative cerebral artery territory analysis

In 174 patients with MMD, the values of the ratio of CBF of the supply territories of ACA, MCA, and PCA to ipsilateral cerebellar hemispheres were calculated before revascularization. The correlations between DSC-CBF_relative_ and ASL-CBF_relative_ (1,500 ms) were statistically significant except for the correlations on the blood supply areas of ACA (right) and MCA (right). The correlations between DSC-CBF_relative_ and ASL-CBF_relative_ (2,500 ms) were statistically significant except for the correlation on the blood supply areas of PCA (right) (see Table 2).

**TABLE 2:** The correlation between dynamic susceptibility contrast-cerebral blood flow (DSC-CBF) and arterial spin labeling (ASL)-CBF before revascularization.

Vascular territories	ASL-CBF_relative_	DSC-CBF_relative_	r	*P*-values
ACA (left)	1,500 ms	DSC	0.221	0.003
ACA (left)	2,500 ms	DSC	0.438	0.000
ACA (right)	1,500 ms	DSC	0.044	0.567
ACA (right)	2,500 ms	DSC	0.23	0.002
MCA (left)	1,500 ms	DSC	0.325	0.000
MCA (left)	2,500 ms	DSC	0.225	0.003
MCA (right)	1,500 ms	DSC	0.112	0.141
MCA (right)	2,500 ms	DSC	0.264	0.000
PCA (left)	1,500 ms	DSC	0.268	0.000
PCA (left)	2,500 ms	DSC	0.212	0.005
PCA (right)	1,500 ms	DSC	0.245	0.001
PCA (right)	2,500 ms	DSC	0.076	0.319

**FIGURE 1:**
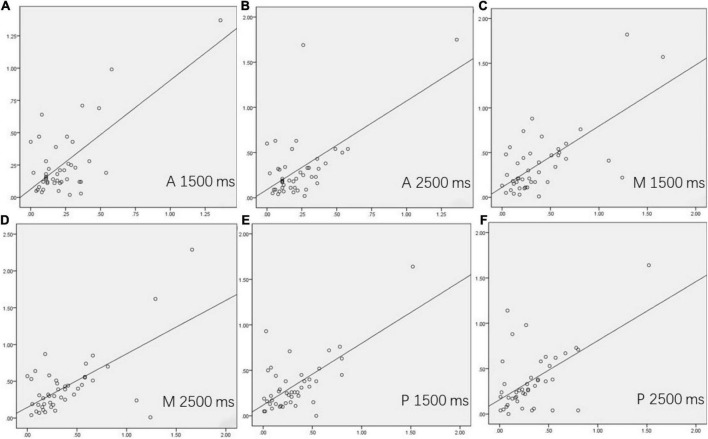
The correlations between dynamic susceptibility contrast-cerebral blood flow (DSC-CBF)_minus_ and arterial spin labeling (ASL)-CBF_minus_ (1,500 ms) and between DSC-CBF_minus_ and ASL-CBF_minus_ (2,500 ms) in 44 surgical patients. **(A–F)** showed the correlation of the supply territories of ACA, MCA, and PCA between DSC-CBF_minus_ and ASL-CBF_minus_ (1,500 ms) and between DSC-CBF_minus_ and ASL-CBF_minus_ (2,500 ms) on the surgical side.

For 44 patients with MMD who underwent unilateral surgery, the values of the difference between the postoperative ratios of CBF and preoperative ratios of CBF were calculated on the supply territories of ACA, MCA, and PCA. All correlations between DSC-CBF_minus_ and ASL-CBF_minus_ (1,500 ms) and between DSC-CBF_minus_ and ASL-CBF_minus_ (2,500 ms) were statistically significant (see Table 3 and Figure 1).

**TABLE 3:** The correlation between dynamic susceptibility contrast-cerebral blood flow (DSC-CBF) and arterial spin labeling (ASL)-CBF after revascularization.

Vascular territories	ASL-CBF_minus_	DSC-CBF_minus_	r	*P*-values
ACA (surgical side)	1,500 ms	DSC	0.306	0.044
ACA (surgical side)	2,500 ms	DSC	0.366	0.014
MCA (surgical side)	1,500 ms	DSC	0.517	0.000
MCA (surgical side)	2,500 ms	DSC	0.409	0.006
PCA (surgical side)	1,500 ms	DSC	0.424	0.004
PCA (surgical side)	2,500 ms	DSC	0.400	0.007

### Quantitative alberta stroke program early CT score areas analysis

**FIGURE 2:**
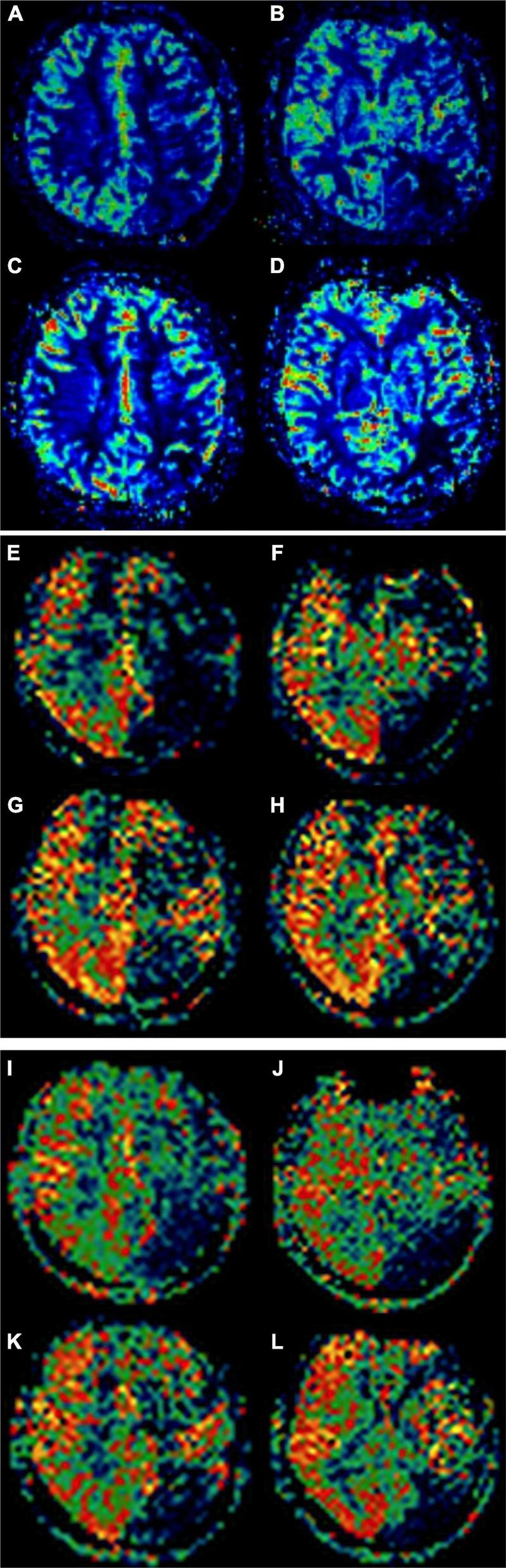
A 51-year-old female patient with moyamoya disease (MMD) underwent encephaloduroarteriosynangiosis (EDAS) on the left side. Alberta Stroke Program Early CT Score (ASPECTS) area ischemia scores of dynamic susceptibility contrast-cerebral blood flow (DSC-CBF) before revascularization were 5 points **(A,B)**. ASPECTS area ischemia scores after revascularization were 8 points **(C,D)**. ASPECTS area ischemia scores of arterial spin labeling (ASL)-CBF (1,500 ms) before revascularization were 5 points **(E,F)**. ASPECTS area ischemia scores after revascularization were 9 points **(G,H)**. ASPECTS area ischemia scores of ASL-CBF (2,500 ms) before revascularization was 4 points **(I,J)**. ASPECTS area ischemia scores after revascularization were 8 points **(K,L)**.

The consistency of ASL-CBF (1,500 ms), ASL-CBF (2,500 ms), and DSC-CBF in assessing ASPECTS area ischemia scores before and after revascularization was good for calculating the ICC. The result of the ICC of ASL-CBF (1,500 ms), ASL-CBF (2,500 ms), and DSC-CBF in assessing ASPECTS area ischemia scores before revascularization was 0.7696 (0.6200–0.8666) and the result of the ICC of ASL-CBF (1,500 ms), ASL-CBF (2,500 ms), and DSC-CBF after revascularization was 0.8335 (0.7255–0.9036). The differences in the preoperative ASPECTS area ischemia scores of ASL-CBF (1,500 ms), ASL-CBF (2,500 ms), and DSC-CBF were not statistically significant (*F* = 2.523, *p* = 0.084), and the differences of the postoperative ASPECTS area ischemia scores were not also statistically significant (*F* = 2.146, *p* = 0.121). The postoperative ASPECTS area ischemia scores were higher than the preoperative scores using all three methods and the differences were statistically significant (*p* < 0.05) (see Table 4 and Figure 2). This indicated that cerebral ischemia was improved after revascularization.

**TABLE 4:** Comparison Alberta Stroke Program Early CT Score (ASPECTS) area ischemia scores before and after revascularization.

CBF	Before revascularization	After revascularization	*t*	*P*-values
ASL-CBF (1,500 ms)	5.1 ± 3.3	7.9 ± 2.2	−3.052	0.003
ASL-CBF (2,500 ms)	4.8 ± 3.3	7.5 ± 2.3	−4.415	0.000
DSC-CBF	5.2 ± 2.1	8.5 ± 2.4	−4.122	0.000

## Discussion

In this study, we analyzed the correlation between DSC-CBF_relative_ and ASL-CBF_relative_ (1,500 ms) and between DSC-CBF_relative_ and ASL-CBF_relative_ (2,500 ms) of the main arterial supply areas in 174 patients with MMD before surgery. The results showed that there was no significant correlation between DSC-CBF_relative_ and ASL-CBF_relative_ (1,500 ms) in only two blood supply areas of all the main arterial supply areas. There was no significant correlation between DSC-CBF_relative_ and ASL-CBF_relative_ (2,500 ms) on only one blood supply area of all the main arterial supply areas. Analysis of preoperative data showed that the correlation between DSC-CBF_relative_ and ASL-CBF_relative_ (2,500 ms) was slightly better than the correlation between DSC-CBF_relative_ and ASL-CBF_relative_ (1,500 ms) in major vascular territories. For forty-four patients with MMD after unilateral surgery, the correlation between DSC-CBF_minus_ and ASL-CBF_minus_ (1,500 ms) and between DSC-CBF_minus_ and ASL-CBF_minus_ (2,500 ms) of the main arterial supply areas on the surgical side was analyzed. The results showed that the correlation of all data on the blood supply areas of ACA, MCA, and PCA was statistically significant. Previous studies in healthy subjects showed a good correlation between CBF measured using the gold standard ^15^O-PET and ASL-CBF (Ye et al., 2000; Wang et al., 2014). The results of this study also showed a clear correlation between DSC-CBF and ASL-CBF. Recent studies showed that PLD is usually chosen between 1,500 and 2,000 ms in the clinical use of pCASL, which can better show true cerebral perfusion (Qiu et al., 2012; Wang et al., 2012; Han et al., 2017; Tong et al., 2020). In this study, however, we chose two PLDs, 1,500 and 2,500 ms. CBF in patients with MMD is very complicated, and we found that the delay time of 2,500 ms was better correlated with DSC and can more accurately assess CBF and collateral circulation in patients with MMD.

MMD is a cerebrovascular disease with progressive stenosis and occlusion of the distal internal carotid artery and the proximal middle cerebral artery with massive collateral circulation. Its cerebral hemodynamics is very complex, but the correct assessment of cerebral perfusion is an important factor in the choice of treatment and prognosis of patients with MMD. DSC cerebral perfusion technique is very common in the clinical application of MMD, and it can provide four parameters; the importance of its value has been fully affirmed in clinical practice. The combination of DSC-CBF with rMTT and TTP can accurately identify the areas of abnormal cerebral hypoperfusion in patients with MMD before operation and evaluate the changes in cerebral perfusion after the operation (Hirai et al., 2017). The disadvantages of DSC are traumatic examination, the need for injection of contrast, and semiquantitative measurements. ASL cerebral perfusion techniques are increasingly used in clinical practice because the advantages are that it does not require the injection of contrast agents, can be repeated, and can be completely quantified. The importance of ASL has been well validated in the study of ischemic brain diseases, and reduced CBF can cause irreversible damage to brain tissue, thereby increasing the risk of stroke (Noguchi et al., 2013; Soman et al., 2020).

For patients with MMD, accurate assessment of cerebral perfusion before and after surgery is very important to evaluate the therapeutic effect and prognosis. In this study, cerebral perfusion was assessed through preoperative and postoperative ASPECTS area ischemia scores. The consistency of preoperative and postoperative ASPECTS area ischemia scores of the assessment of DSC-CBF, ASL-CBF (PLD = 1,500 ms), and ASL-CBF (PLD = 2,500 ms) was good. In addition, the difference of preoperative ASPECTS area ischemia scores between DSC-CBF, ASL-CBF (PLD = 1,500 ms), and ASL-CBF (PLD = 2,500 ms) and the difference of postoperative ASPECTS area ischemia scores between DSC-CBF, ASL-CBF (PLD = 1,500 ms), and ASL-CBF (PLD = 2,500 ms) were not statistically significant (*p* > 0.05). The differences between preoperative and postoperative ASPECTS area ischemia scores using these three methods of DSC-CBF, ASL-CBF (PLD = 1,500 ms), and ASL-CBF (PLD = 2,500 ms) were statistically significant, and the postoperative scores were all greater than the preoperative scores. This suggests that ASL and DSC had consistent results in assessing preoperative and postoperative hypoperfusion areas of ASPECTS areas in patients with MMD and that both can accurately assess changes in postoperative CBF. Therefore, ASL was an imaging method that can replace DSC to evaluate cerebral perfusion changes before and after surgery in patients with MMD.

This study has several limitations. First, 3D pCASL with two single PLD times and only one parameter of CBF was compared with DSC-CBF. A subsequent comparative analysis of multi-delay ASL and DSC will be performed. Second, this study used the ratio of ASL-CBF and DSC-CBF to the CBF of the ipsilateral cerebellar hemisphere for correlation analysis, without direct use of ASL-CBF quantitative parameter values. Finally, for patients with MMD, ASL (PLD = 2,500 ms) can more accurately show cerebral blood perfusion, and further study with larger samples is needed.

## Conclusion

In this study, there was a significant correlation between ASL-CBF and DSC-CBF in patients with MMD. Both ASL-CBF and DSC-CBF can evaluate cerebral perfusion before and after surgery. ASL, as a cerebral perfusion examination method without a contrast agent, can replace DSC as an imaging method for the evaluation of cerebral perfusion in patients with MMD.

## Data availability statement

The raw data supporting the conclusions of this article will be made available by the authors, without undue reservation.

## Ethics statement

This study has been approved by the appropriate Ethics Committee of the Chinese PLA General Hospital. Written informed consent to participate in this study was provided by the participants’ legal guardian/next of kin. Written informed consent was obtained from the individual(s), and minor(s)’ legal guardian/next of kin, for the publication of any potentially identifiable images or data included in this article.

## Author contributions

HZ drafted the manuscript. JC and CH conceived and designed the study. ML and FS analyzed the data. SL, DL, and XS were responsible for the collection of data and image analysis. All authors contributed to the article and approved the submitted version.

## Supplementary material

Supplementary Image 1 Correlative scatter plots of the supply territories of ACA-left between DSC-CBF_relative_ and ASL-CBF_relative_ (1500 ms) before revascularization.

Supplementary Image 2 Correlative scatter plots of the supply territories of ACA-left between DSC-CBF_relative_ and ASL-CBF_relative_ (2500 ms) before revascularization.

Supplementary Image 3 Correlative scatter plots of the supply territories of ACA-right between DSC-CBF_relative_ and ASL-CBF_relative_ (1500 ms) before revascularization.

Supplementary Image 4 Correlative scatter plots of the supply territories of ACA- right between DSC-CBF_relative_ and ASL-CBF_relative_ (2500 ms) before revascularization.

Supplementary Image 5 Correlative scatter plots of the supply territories of MCA-left between DSC-CBF_relative_ and ASL-CBF_relative_ (1500 ms) before revascularization.

Supplementary Image 6 Correlative scatter plots of the supply territories of MCA-left between DSC-CBF_relative_ and ASL-CBF_relative_ (2500 ms) before revascularization.

Supplementary Image 7 Correlative scatter plots of the supply territories of MCA-right between DSC-CBF_relative_ and ASL-CBF_relative_ (1500 ms) before revascularization.

Supplementary Image 8 Correlative scatter plots of the supply territories of MCA-right between DSC-CBF_relative_ and ASL-CBF_relative_ (2500 ms) before revascularization.

Supplementary Image 9 Correlative scatter plots of the supply territories of PCA-left between DSC-CBF_relative_ and ASL-CBF_relative_ (1500 ms) before revascularization.

Supplementary Image 10 Correlative scatter plots of the supply territories of PCA-left between DSC-CBF_relative_ and ASL-CBF_relative_ (2500 ms) before revascularization.

Supplementary Image 11 Correlative scatter plots of the supply territories of PCA-right between DSC-CBF_relative_ and ASL-CBF_relative_ (1500 ms) before revascularization.

Supplementary Image 12 Correlative scatter plots of the supply territories of PCA-right between DSC-CBF_relative_ and ASL-CBF_relative_ (2500 ms) before revascularization.
