# Supplementary figures and images for: The value of 3D pseudo-continuousarterial spin labeling perfusion imaging in moyamoya disease—Comparison with dynamic susceptibility contrast perfusion imaging
Source: Front Neurosci. 2022 Aug 5;16:944246. doi: 10.3389/fnins.2022.944246 (PMC9389231; doi:10.3389/fnins.2022.944246)

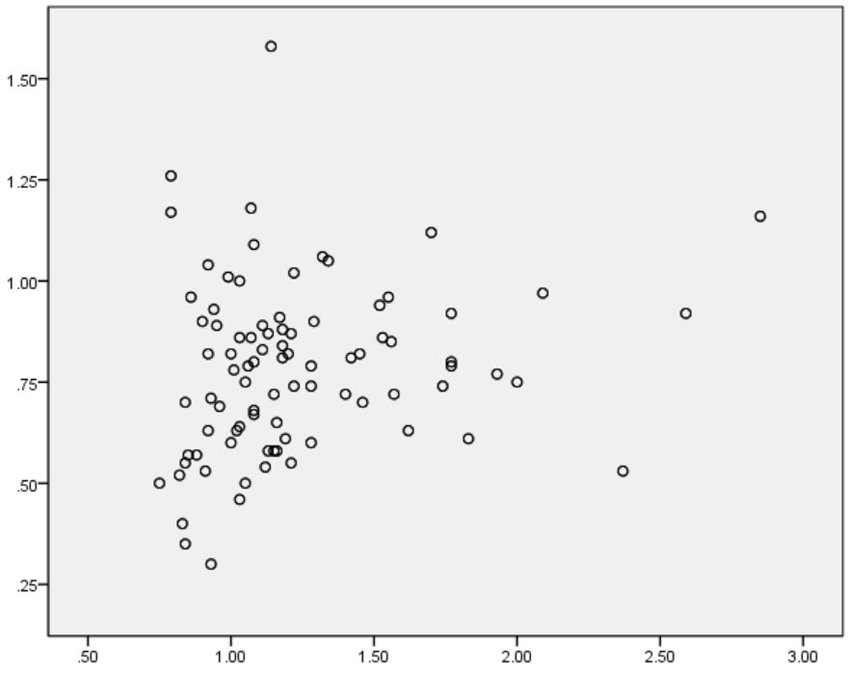

Supplement: Supplementary Image 1 — Correlative scatter plots of the supply territories of ACA-left between DSC-CBFrelative and ASL-CBFrelative (1500 ms) before revascularization. [file Image_1.JPEG]

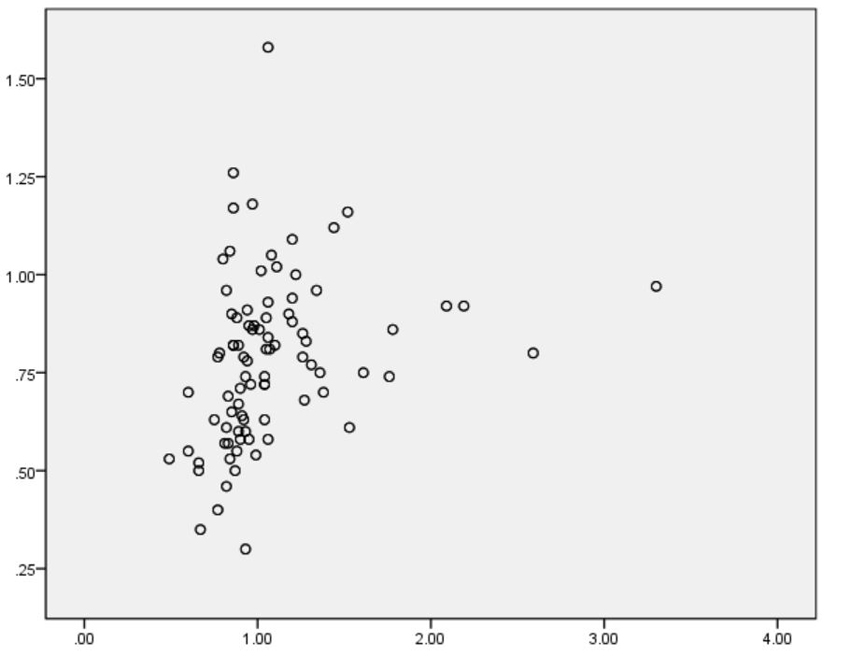

Supplement: Supplementary Image 2 — Correlative scatter plots of the supply territories of ACA-left between DSC-CBFrelative and ASL-CBFrelative (2500 ms) before revascularization. [file Image_2.JPEG]

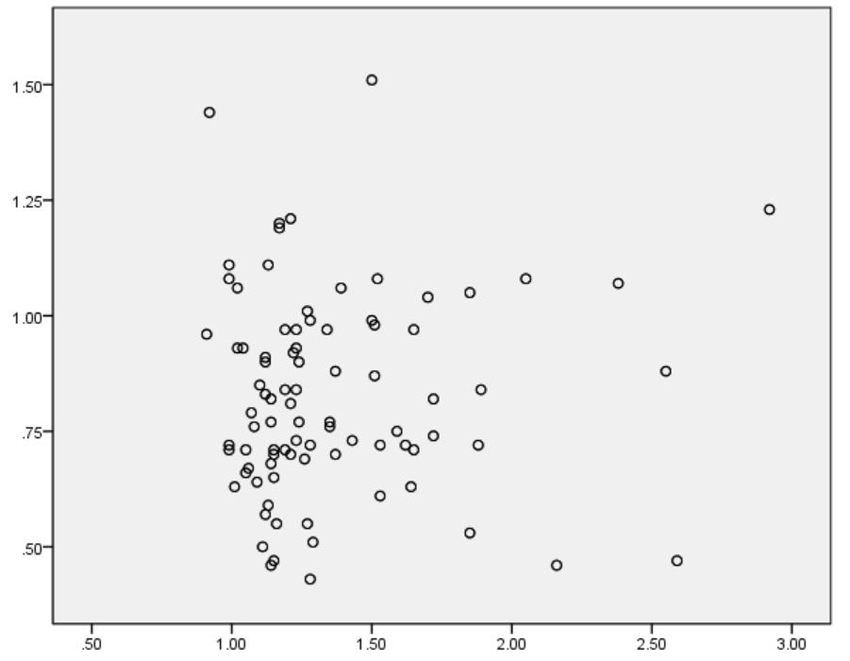

Supplement: Supplementary Image 3 — Correlative scatter plots of the supply territories of ACA-right between DSC-CBFrelative and ASL-CBFrelative (1500 ms) before revascularization. [file Image_3.JPEG]

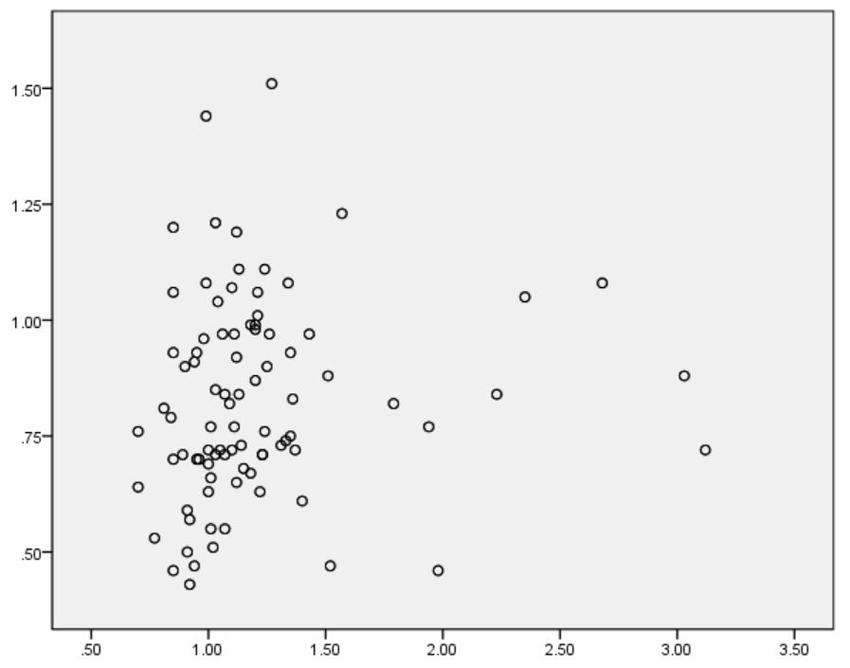

Supplement: Supplementary Image 4 — Correlative scatter plots of the supply territories of ACA- right between DSC-CBFrelative and ASL-CBFrelative (2500 ms) before revascularization. [file Image_4.JPEG]

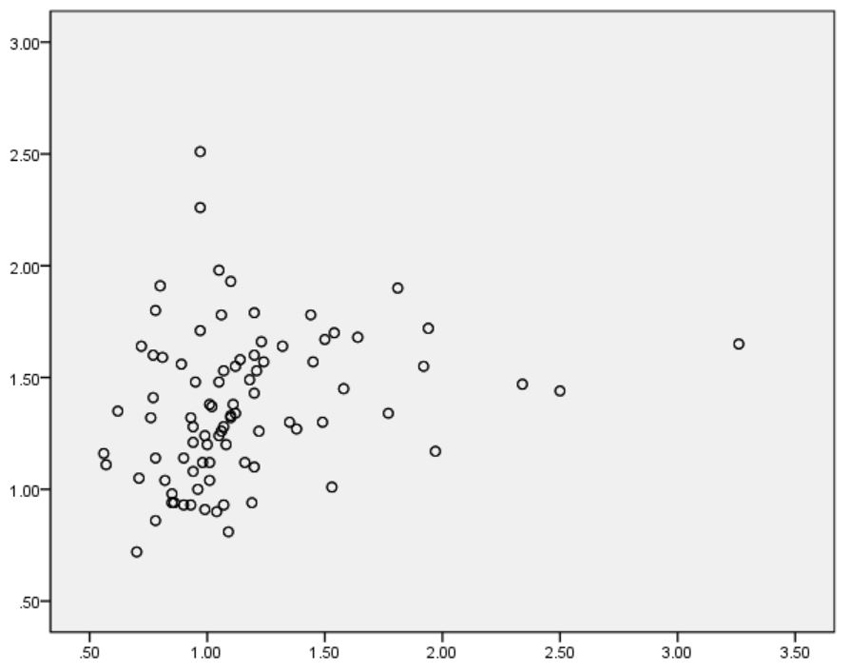

Supplement: Supplementary Image 5 — Correlative scatter plots of the supply territories of MCA-left between DSC-CBFrelative and ASL-CBFrelative (1500 ms) before revascularization. [file Image_5.JPEG]

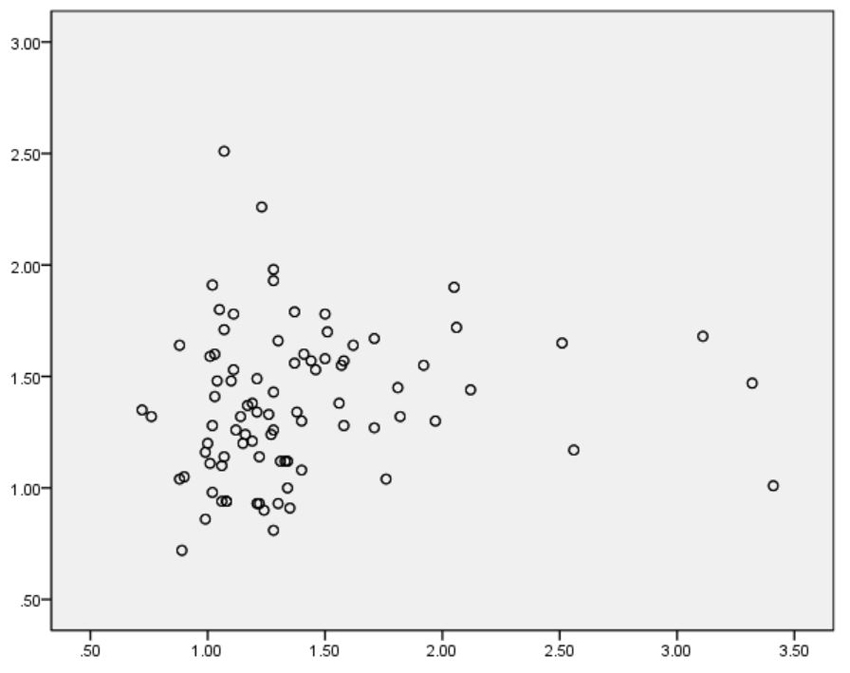

Supplement: Supplementary Image 6 — Correlative scatter plots of the supply territories of MCA-left between DSC-CBFrelative and ASL-CBFrelative (2500 ms) before revascularization. [file Image_6.JPEG]

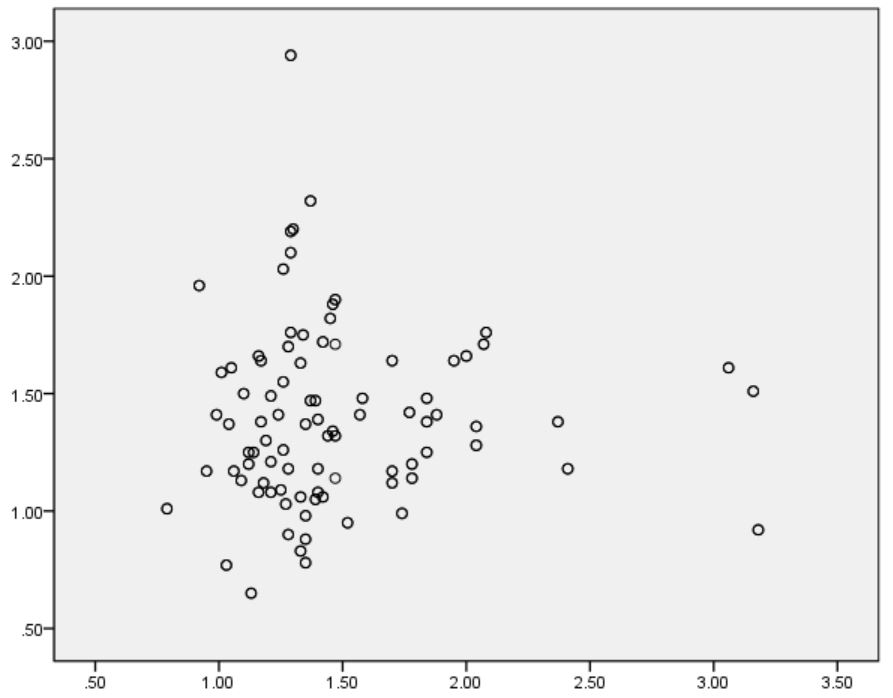

Supplement: Supplementary Image 7 — Correlative scatter plots of the supply territories of MCA-right between DSC-CBFrelative and ASL-CBFrelative (1500 ms) before revascularization. [file Image_7.JPEG]

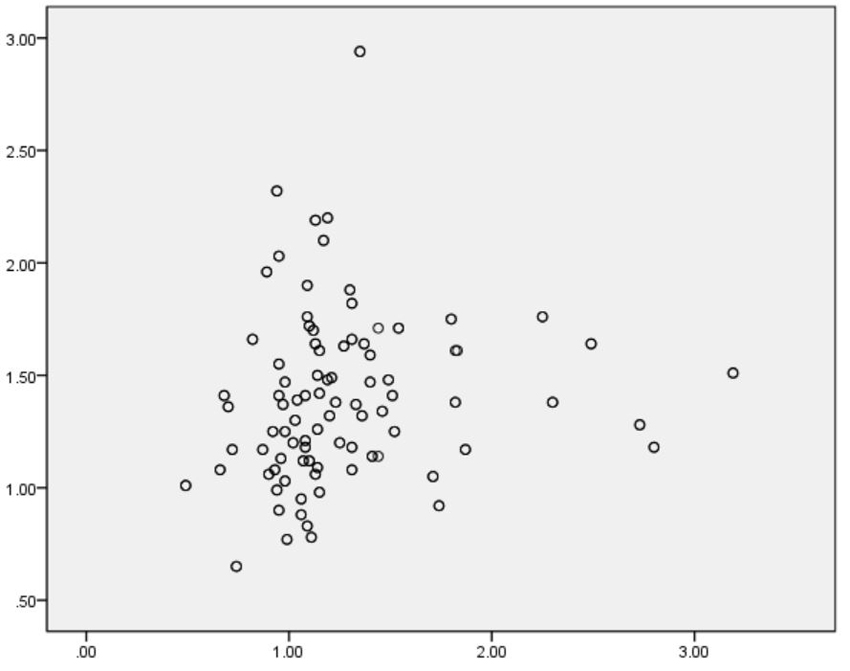

Supplement: Supplementary Image 8 — Correlative scatter plots of the supply territories of MCA-right between DSC-CBFrelative and ASL-CBFrelative (2500 ms) before revascularization. [file Image_8.JPEG]

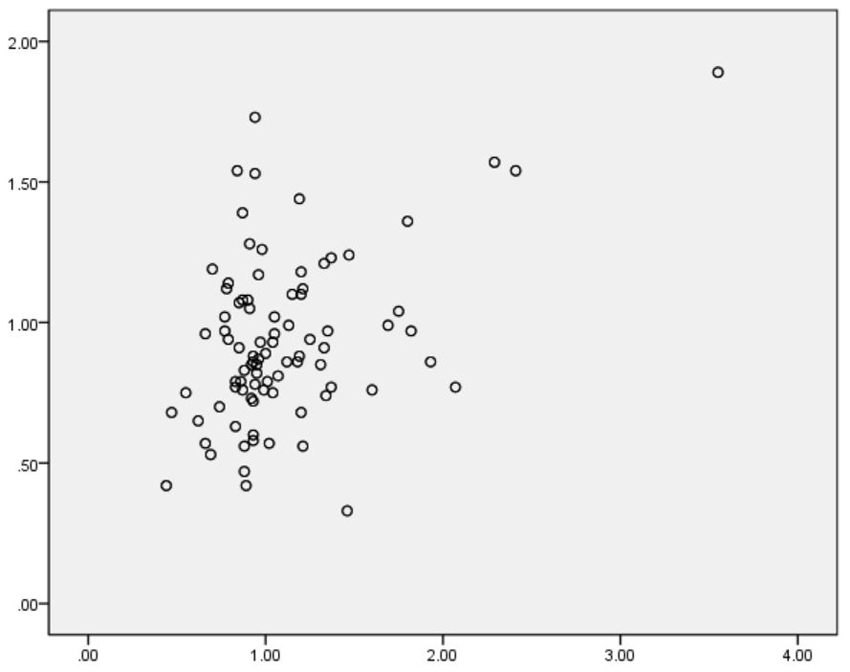

Supplement: Supplementary Image 9 — Correlative scatter plots of the supply territories of PCA-left between DSC-CBFrelative and ASL-CBFrelative (1500 ms) before revascularization. [file Image_9.JPEG]

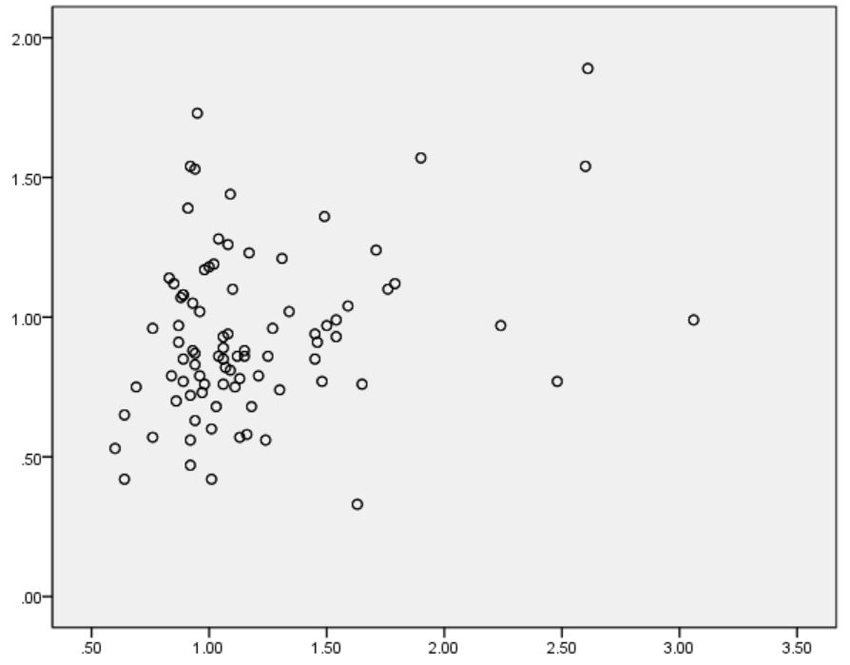

Supplement: Supplementary Image 10 — Correlative scatter plots of the supply territories of PCA-left between DSC-CBFrelative and ASL-CBFrelative (2500 ms) before revascularization. [file Image_10.JPEG]

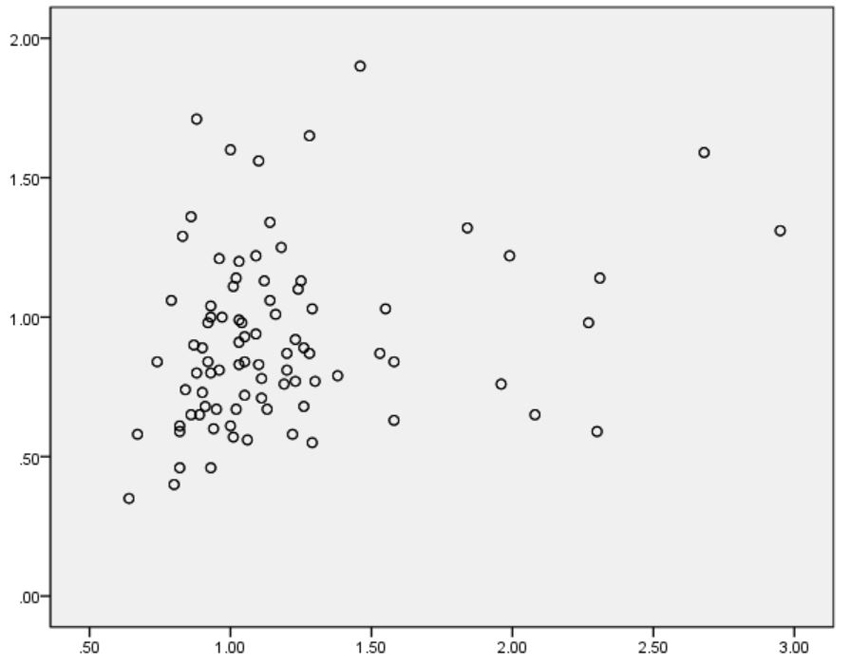

Supplement: Supplementary Image 11 — Correlative scatter plots of the supply territories of PCA-right between DSC-CBFrelative and ASL-CBFrelative (1500 ms) before revascularization. [file Image_11.JPEG]

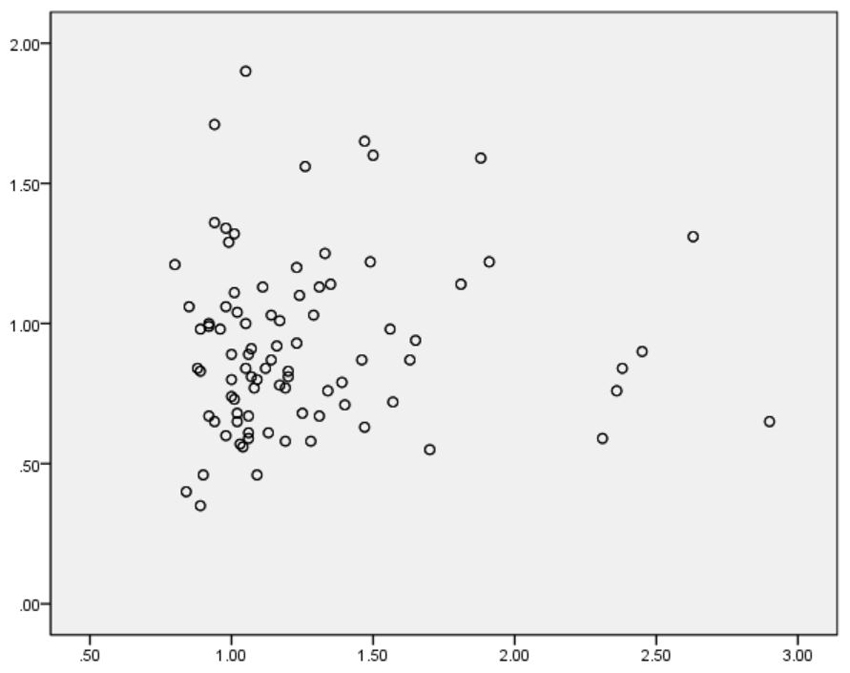

Supplement: Supplementary Image 12 — Correlative scatter plots of the supply territories of PCA-right between DSC-CBFrelative and ASL-CBFrelative (2500 ms) before revascularization. [file Image_12.JPEG]
